# A large screen identifies beta-lactam antibiotics which can be repurposed to target the syphilis agent

**DOI:** 10.1038/s44259-023-00006-3

**Published:** 2023-06-01

**Authors:** Kathryn A. Hayes, Jules M. Dressler, Steven J. Norris, Diane G. Edmondson, Brandon L. Jutras

**Affiliations:** 1 Translational Biology, Medicine, and Health, Virginia Tech, Blacksburg, VA 24061 USA; 2 Fralin Life Sciences Institute, Virginia Tech, Blacksburg, VA 24061 USA; 3 Department of Biochemistry, Virginia Tech, Blacksburg, VA 24061 USA; 4 Department of Pathology and Laboratory Medicine, University of Texas Health Science Center at Houston, Houston, TX USA; 5 Department of Microbiology and Molecular Genetics, McGovern Medical School, University of Texas Health Science Center at Houston, Houston, TX USA; 6 Center for Emerging, Zoonotic and Arthropod-borne Pathogens, Virginia Tech, Blacksburg, VA 24061 USA

**Keywords:** Glycobiology, Bacteriology

## Abstract

Syphilis, caused by the spirochete *Treponema pallidum* subsp. *pallidum* (hereafter called *T. pallidum*), is re-emerging as a worldwide sexually transmitted infection. A single intramuscular dose of benzathine penicillin G is the preferred syphilis treatment option. Both supply shortage concerns and the potential for acquired antibiotic resistance further the need to broaden the repertoire of syphilis therapeutics. We reasoned that other β-lactams may be equally or more effective at targeting the disease-causing agent, *Treponema pallidum*, but have yet to be discovered due to a previous lack of a continuous in vitro culture system. Recent technical advances with respect to in vitro *T. pallidum* propagation allowed us to conduct a high-throughput screen of almost 100 β-lactams. Using several molecular and cellular approaches that we developed or adapted, we identified and confirmed the efficacy of several β-lactams that were similar to or outperformed the current standard, benzathine penicillin G. These options are either currently used to treat bacterial infections or are synthetic derivatives of naturally occurring compounds. Our studies not only identified additional potential therapeutics in the resolution of syphilis, but provide techniques to study the complex biology of *T. pallidum*—a spirochete that has plagued human health for centuries.

## Introduction

Syphilis is an escalating, worldwide sexually transmitted infection that poses a major threat to human health. Despite an active initiative by the World Health Organization to lower cases, more than 7 million new cases were reported in 2020 globally, an increase of 12.7% over 4 years^1–4^. Over the same period of time, the United States experienced a 52% increase in cases. Congenital syphilis—arising from gestational transmission from mother to child—increased 2.9-fold from 2015 to 2019^5^. While men who have sex with men and female sex workers have high reported annual incidences of 7.5% and 14%, respectively^1,2^, the infection rate in heterosexual populations is also increasing. All data and trends suggest that syphilis will continue to be a global problem much like it has been for the better part of the past five centuries.

The clinical symptoms of syphilis vary widely in type and severity, earning it the moniker ‘*the great imitator*’^6^. Symptoms generally appear within three weeks with the occurrence of the characteristic chancre lesion at the site of inoculation^7,8^. If this primary stage is not treated promptly and properly, the secondary and tertiary stage may follow resulting in a multisystem infection. Later stages of the disease cause irreversible damage to several organs and can be fatal^9–11^. Congenital syphilis may occur at any point in pregnancy and is the second leading cause of preventable stillbirth in the United States^12^.

Despite the historical significance of syphilis, little is known about the disease-causing agent, *Treponema pallidum*. Our understanding of *T. pallidum*, and by extension syphilis, has been hindered by the lack of a faithful, prolonged in vitro cultivation system. In the early 1980s *T. pallidum* propagation was reported, but subcultures could not be maintained^13^. A watershed moment occurred when long-term, in vitro growth was achieved, and the culture strategy did not impact cell viability nor infectivity^14–16^. This breakthrough has provided many new avenues of research—from genetic tools to new potential therapeutic strategies^17–21^.

The primary treatment option for early syphilis is a single intramuscular dose of the β-lactam benzathine penicillin G (also known as benzathine penicillin)^22–24^, which could pose several problems. Approximately 1–10% of the human population is estimated to have a penicillin allergy^25,26^. These allergic reactions can range in both severity and degree of cross-reactivity with other β-lactams. In these instances, patients are often micro-dosed with penicillin until the allergy is overcome^27,28^. Supply shortages have been impacting penicillin availability since the mid-2010 s, leading to the use of antibiotics with unknown efficacy^29,30^. This ill-advised practice can lead to incomplete bacterial clearance or even acquired antibiotic resistance, as was the case with azithromycin and erythromycin^31–33^. Finally, while *T. pallidum* has remained susceptible, it is unwise to assume that antibiotic resistance to penicillin is impossible^31^. Doxycycline is the only other recommended alternative treatment, but it (1) requires multiple doses over 2–4 weeks; (2) is not effective against later stages of disease, and (3) cannot be prescribed to pregnant individuals^22^.

We reasoned that there are other β-lactams with similar or greater efficacy against *T. pallidum*, relative to benzathine penicillin G, but have yet to be discovered. Here, we report the first large-scale drug screen of β-lactams and determine their relative efficacy against *T. pallidum* growth in vitro. Using a series of molecular and cellular approaches, we have identified and confirmed several candidate β-lactams that could be effective in treating syphilis.

## Results

### Cell cycle dynamics of *T. pallidum* in culture

**Fig. 1:**
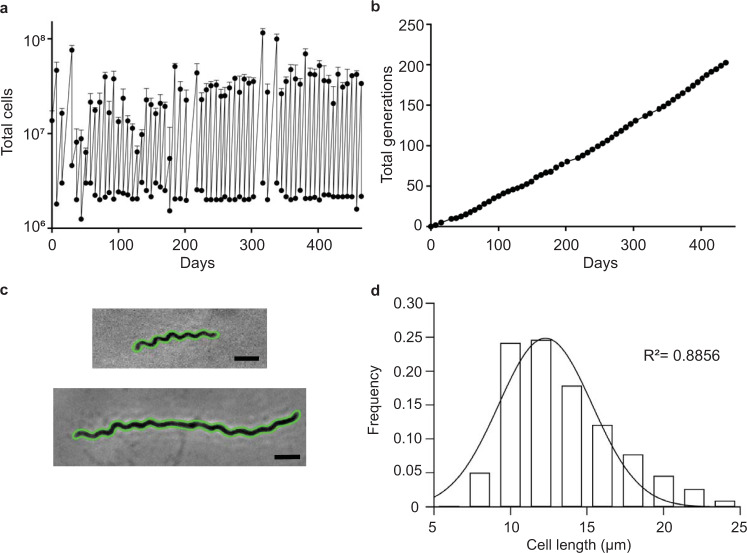
Growth kinetics and characteristics of in vitro cultivated *T*. *pallidum*. **a** Sawtooth plot of continual in vitro *T. pallidum* multiplication. The final cell density of six independent cultures was determined and the average (±s.d.) are shown. At each time point, a fraction of the harvested cells were used to inoculate fresh media. **b** Cumulative generations achieved during in vitro *T. pallidum* cultivation. **c** Automated cell detection of phase-contrast micrographs. Fixed cells were imaged by phase-contrast microscopy and the automated cell detection software Oufti^39^ successfully identified the boundaries (green) of each cell. Cells representing the beginning (above) and end (below) of the *T. pallidum* cell cycle are shown. Scale bars 2 µm. **d** Population-level analysis of individual cell length measurements. Data collected from cell meshes (**c**) was used to determine the cell length distribution of 414 cells and fit to a Gaussian distribution (line, R^2^ = 0.8856).

Prolonged in vitro *T. pallidum* cultivation was recently described and successfully adopted by at least two other research groups to date^14,17,34^. Our laboratory has been cultivating *T. pallidum* subsp. *pallidum*, strain Nichols for intermittent periods over the past 3 years. Prior to starting our analysis of β-lactams, we sought to attain continuous growth and characterize the growth dynamics of our laboratory’s in vitro *T. pallidum* culture. *T. pallidum* were co-cultured with rabbit epithelial cells (Sf1Ep) in a 1.5% O_2_, 5% CO_2_ environment with TpCM2 media as described by Edmondson et al.^14^ Every seven days bacterial cell density was determined by microscopic enumeration and a fraction was used to seed new cultures, creating a sawtooth plot (Fig. 1a). Our most recent continuous culture, which was used for all studies described below, routinely reached densities comparable to those previously reported and had a mean doubling time of 52.3 (±13.2) hours (Fig. 1a)^14^. Since culture inception, we have achieved over 100 total cumulative generations (Fig. 1b) and have maintained continual growth for over a year (Fig. 1a).

All spirochetes studied to date divide by binary fission^35–38^. Binary fission requires that a mother cell double in cell length and divide in the middle, producing two equally sized daughter cells. By measuring the length of each cell in a population, we are able to determine if our culture is being maintained under static conditions. The latter is an important feature in understanding relative drug efficacy and can provide further information about rate-limiting steps during *T. pallidum* propagation in vitro. We determined the in vitro cell length distribution of *T. pallidum* by imaging fixed cells acquired by phase-contrast microscopy. Cells were fixed to prevent aberrations that may occur upon air exposure. Population-level analysis of individual cells was facilitated by Oufti, an automated cell detection platform that is capable of measuring several morphological features at sub-pixel resolution^39^. With parameterization, Oufti was able to accurately identify cell boundaries in phase-contrast micrographs (Fig. 1c). Analysis of >400 cells indicated that in vitro cultivated *T. pallidum* achieved a mean cell length of 13.31 ± 3.51 µm (Fig. 1d), similar to those propagated in vivo^40^. The cell length distribution was normal (*r*^2^ = 0.8856) but had a slight skew towards longer cells (Fig. 1d). From these data we conclude that our culture conditions produce continuous growth and stable growth kinetics which are in line with those achieved in other laboratories^14^. Thus, we proceeded to use this system to assess *T. pallidum* susceptibility to various antibiotics.

### Comparative screen of Beta-lactam efficacy in vitro

Penicillins and cephalosporins are types of β-lactams, a class of antibiotics that function by irreversibly binding to penicillin-binding proteins (PBPs). This prevents peptidoglycan (PG) synthesis and remodeling, leading to cell death. The efficacy of a particular type of β-lactam to treat a bacterial infection is largely based on outer membrane penetrance and the affinity for one or more essential PBPs in PG synthesis^41–43^. While the molecular players involved in *T. pallidum* PG biosynthesis and their function(s) are still being elucidated, benzathine penicillin G clearly satisfies both requirements^44–46^. We reasoned that one or more other β-lactams that are both commercially available and approved for use in humans may be active against *T. pallidum* at similar concentrations to benzathine penicillin G, and that the newly established culture system provides an opportunity to test our hypothesis.

**Fig. 2:**
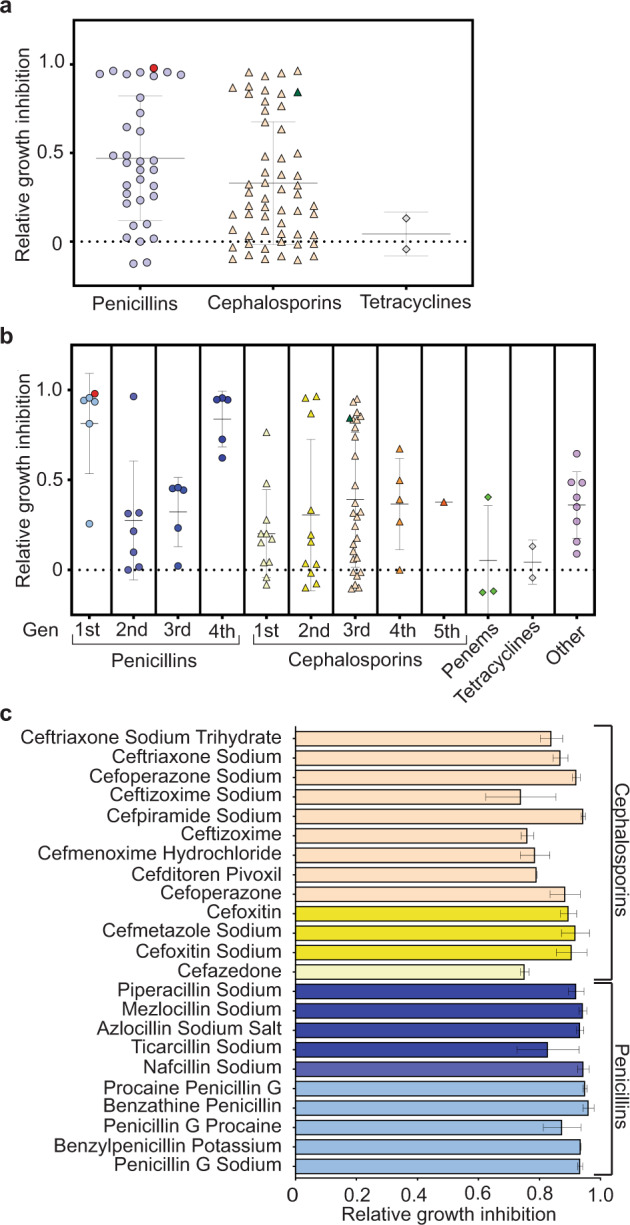
Large-scale analysis of β-lactam growth inhibition. **a** The relative efficacy of 89 different β-lactams was assessed at a final concentration of 5 nM against in vitro cultured *T. pallidum* and grouped based on classification. Growth inhibition was determined by the percentage of cells that remained after treatment, relative to untreated control cultures treated with the appropriate diluent (water or DMSO). *Other* antibiotics included Aztrenoam, Sulbeniciillin sodium, Sultamicillin, Pevmecillinam hydrochloride, Adminocillin, 6-aminopenicillanic acid, 7-Aminocephalosporanic acid, Ceftiofur (3rd generation, but only used in veterinary medicine). *Penems* included Faropenem, Doripenem, and Ertapenem. Doxycycline and tetracycline were included for comparative purposes. Red, and green data points represent benzathine penicillin G and ceftriaxone, respectively. A complete list of the antibiotic panel can be found in Supplementary Table 1. **b** Data collected in **a** were further broken down by generation. Red, and green data points represent benzathine penicillin G and ceftriaxone, respectively. **c** The top 25% of compounds tested in **a**, **b** were re-evaluated on separate cultures and batches of media and grouped based on class. Data shown represent mean values (±s.d.) of two independent cultures.

We determined the relative in vitro efficacy of 89 β-lactams found in a commercially available compound library (see methods and Supplementary Table 1). Each compound was tested at a final concentration of 5 nM and the relative efficiency was determined by comparative studies in untreated control cultures, supplemented with the same amount of diluent (water or DMSO, Supplementary Table 1). We chose this concentration based on estimates of the effective concentration range of benzathine penicillin G^47^. After one week of incubation with an active *T. pallidum* culture, cells were harvested and enumerated. In general, penicillins were slightly more effective at killing *T. pallidum* relative to cephalosporins, but both significantly outperformed the two tetracyclines tested (Fig. 2a). We note that while doxycycline has been shown to kill *T. pallidum*, but it is not effective at 5 nM (Fig. 2a)^20^. Relative efficacy differences notwithstanding, both groups of β-lactams were highly variable (Fig. 2a), which is to be expected for a 5 nM dose. We further broke down each group by generation and found striking trends. The first and fourth generations of penicillin were significantly better at eradicating in vitro cultivated *T. pallidum* than second and third generations (Fig. 2b). This is noteworthy and provides further credence to our results since fourth generation penicillins are the synthetic analogs of the first generation and share similar molecular characteristics. In contrast, generations of cephalosporins are loosely separated by their spectrum of activity and they did not produce any apparent difference, but several were indeed effective (Fig. 2a, b). To further examine the validity of our initial findings, we included other cell wall synthesis inhibitors that have been shown to be effective against different clades of gram-negative bacteria (Supplementary Table 1, Fig. 2b, and refs. ^48,49^). None of the compounds tested under our experimental conditions achieved results comparable to top-performing penicillins and cephalosporins (Fig. 2b).

Our initial screen yielded many promising candidates (Fig. 2a, b), which we went on to analyze further. In an entirely separate set of studies, we sought to confirm the activity of the top 25% of compounds screened (23 candidates); all of which were > 70% effective at preventing growth in our initial screen. Analysis of an additional biological replicate of these select β-lactams confirmed their relative efficacy and produced nearly identical results (Fig. 2c).

**Fig. 3:**
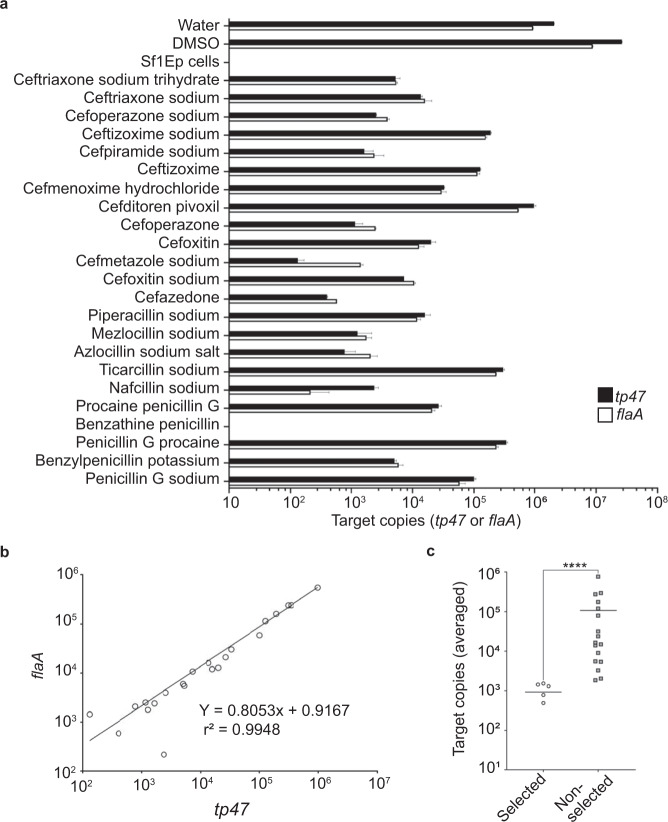
Transcriptional analysis of top-performing β-lactams. **a** RNA was extracted and purified from *T. pallidum* cultures treated with 5 nM of the 23 β-lactams identified in Fig. 2c and analyzed by qRT-PCR using two targets: *flaA* (white) and *tp47* (black). Purified genomic DNA and delta Ct values were used to calculate the number of copies of each target present in each treatment. Post amplification, melting curves were used to confirm the validity of the signal for each target. The diluents, water, and DMSO, were included as positive controls and a mono-culture of SfEp1 cells served as a negative control. All samples were assessed in duplicate and the mean (±s.d.) are shown. **b** Correlation analysis of *tp47* and *flaA* qRT-PCR values attained in **a**. **c** The *flaA* and *tp47* values were averaged for each treatment and grouped based on the 25% lowest samples, relative to the remainder. Statistical analysis, Mann–Whitney *U* test (****p < 0.0001).

So far, our analysis of β-lactam efficacy has been focused on cellular responses to treatment—final cell density relative to untreated control cultures—which relies on microscopic enumeration. Microscopic enumeration does not provide high-resolution information regarding potential differences between drug treatments. To further evaluate the top 25% of β-lactams at the molecular level, we purified RNA from treated cells and assessed mRNA levels with the reasoning that dead or dying cells would be less transcriptionally active. The transcript levels of two well-known reference genes were selected: (1) *tp47*, a highly expressed mRNA that encodes for a lipoprotein, and (2) *flaA*, a constitutive transcript that when translated produces the flagellar sheath protein FlaA^45,50^. The latter was included to ensure that the potential function(s) of Tp47 in PG biosynthesis and β-lactamase activity did not impact our results^44–46^. Since *T. pallidum* in vitro growth requires co-culture with Sf1Ep rabbit epithelial cells, RNA from a Sf1Ep mono-culture was included to account for any off target qRT-PCR signal. As expected, Sf1Ep control mono-cultures produced no measurable levels of either transcript (Fig. 3a). Using genomic DNA to create reference standards, we found that drug treatment produced a range of values that varied on the order of 10^5^ target copies, with benzathine penicillin G yielding no detectable signal (Fig. 3a). These data indicate that our strategy was effective at providing high-resolution information about each compound’s efficacy, relative to our previous cellular data. Both *flaA* and *tp47* transcript levels were similar (Fig. 3a) and values obtained from the same sample were significantly correlated (Fig. 3b), indicating that either can be used for our purposes and that our methods were reliable. As such, we combined the values obtained for both, calculated the average, and determined that six different β-lactams (25%) had, on average, a greater than 10-fold difference in total copy number relative to the average of the remaining 17 treatments (Fig. 3c). These six β-lactams were the focus of further analysis.

### A subset of Beta-lactams act at low nM concentrations

**Fig. 4:**
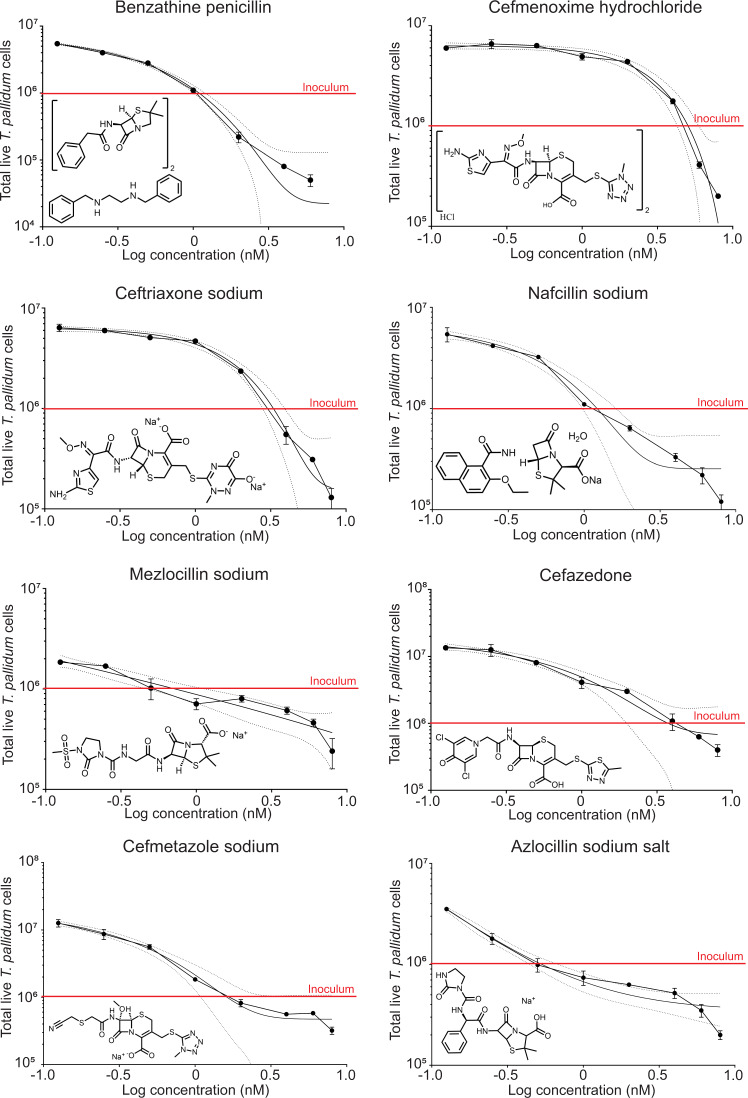
Minimum inhibitory concentration of top-performing β-lactams. The minimum inhibitory concentration of selected compounds (Fig. 3c) was determined by cell enumeration with a serial titration of antibiotic. Values represent the average (±s.d.) of two biological replicates. A standard curve is a modified Gompertz regression with 95% confidence interval shown.

Minimum inhibitory concentration (MIC) calculations are the gold standard for determining the efficiency of a treatment in the context of antimicrobials. Our iterative approach (Figs. 2–3) led to a manageable number of treatment options that could be further evaluated to determine the MIC of top-performing compounds. In addition to the six identified in Fig. 3, we included two additional β-lactams: ceftriaxone sodium and cefmenoxime hydrochloride since (1) the former has been reported to be active against *T. pallidum* at low nM concentrations;^47^ (2) they both produced intermediate-level activity among the top 25%; (3) their cost; and (4) putative half-life (Table 1). Each compound was serially titrated into fresh passages of *T. pallidum*, with two biological replicates for each concentration, and culture density was determined as described earlier (Figs. 2–3). All identified β-lactams were effective and produced MIC values in the low nM range indicating that our iterative sampling approach was successful (Fig. 4 and Table 1), but some differences were also apparent. For instance, azlocillin and mezlocillin produced the lowest MIC values and were ~1.5–2.0 times more effective than benzathine penicillin G (Fig. 4 and Table 1). Furthermore, modified Gompertz regression analysis of azlocillin and mezlocillin treatment clearly indicates improved growth inhibition at lower concentrations (Fig. 4). In addition, the activity of nafcillin in preventing growth was comparable to that of benzathine penicillin G (Fig. 4 and Table 1). Outliers within the top-performing compounds, ceftriaxone, and cefmenoxime, both had ~3-fold higher MICs, which was consistent with our qRT-PCR results (Fig. 3). Nonetheless, all candidates were extremely effective and produced MIC values 10–100 times lower than doxycycline^20^.

**Table 1:** Minimum inhibitory concentration, cost, and half-life of top-performing compounds.

Antibiotic	MIC (nM)	Interpolated MIC (ng/mL)	Cost per gram	Half-life for i.m. ^Ref.^
Benzathine penicillin	1.091	0.992	$3880	6 h^55^
Cefmenoxime hydrochloride	5.047	5.347	$594	0.85–1.15 h^87^
Ceftriaxone sodium	3.296	1.973	$3880	5.4 h^88^
Mezlocillin sodium	0.723	0.406	$1940	0.9 h^89^
Cefazedone	3.750	2.057	$3880	1.85 h^90^
Cefmetazole sodium	1.626	0.802	$3880	1.1–1.4 h^91^
Nafcillin sodium	1.216	0.553	$1940	1.4 h^68^
Azlocillin sodium salt	0.530	0.257	$1940	1.4 h^92^

**Fig. 5:**
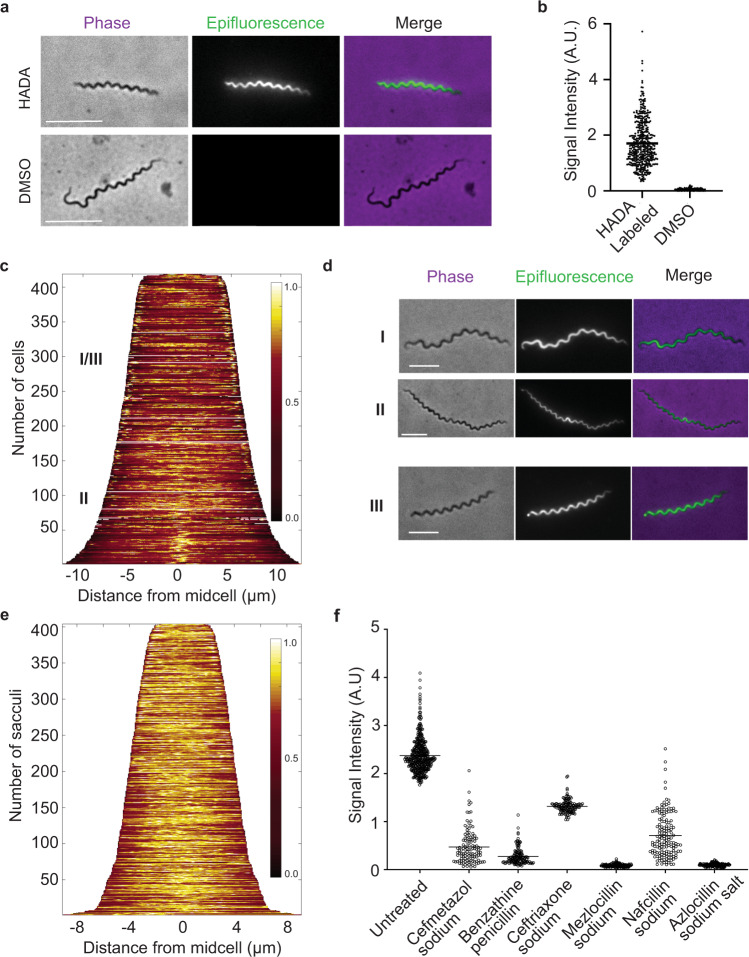
HADA labeling of whole cell and antibiotic-treated sacculi. **a** *T. pallidum* were pulse-labeled with 0.5 mM HADA for 24 h, fixed with paraformaldehyde, and imaged on 2% agarose pads containing PBS. Control cells were exposed to DMSO, the HADA diluent. Scale bar 5 µm. **b** Total fluorescent signal intensity, normalized by cell area, of HADA-labeled (*n* = 414) and unlabeled control cells (*n* = 100). Each dot is a single cell measurement and the mean value is represented by a horizontal line. **c** Demograph showing population-level analysis of HADA-labeled cells organized by length. Heat map represents fluorescent signal intensity in arbitrary units. **d** Example images of cell cycle stages (I-III) observed in HADA-labeled cells. Phase-contrast (left), epifluorescence (center), and merge of the latter two (right) are shown. Stage I: Uniform fluorescence throughout most of the cell cycle. Stage II: Increased HADA signal indicative of cell wall septation (division). Stage III: Increased HADA signal at pole of cell from previous division event. Scale bar 5 µm. **e** Population-level analysis of HADA-derived signal attained from purified peptidoglycan (*n* = 404). Heat map represents fluorescent signal intensity in arbitrary units. **f** Signal intensity of isolated sacculi from growing *T. pallidum* that were exposed to an antibiotic at two times its calculated MIC (Fig. 4) and pulse-labeled with 0.5 mM HADA for 24 h. The total signal intensity of each pixel inside each sacculus was calculated, normalized by area, and the average value of each object is shown as a single dot. The horizonal line represents the mean value of the population. Non-antibiotic-treated control *n* = 404, cefmetazole sodium *n* = 122, benzathine penicillin G *n* = 133, ceftriaxone sodium *n* = 140, azlocillin *n* = 145, mezlocillin *n* = 148; nafcillin *n* = 151.

PBPs, the target of β-lactams, catalyze transglycosylase and transpeptidase reactions required for cell elongation and division, respectively. Muropeptides containing the terminal D-alanine residue(s) are the substrate for the latter. By incubating *T. pallidum* cultures with 7-hydroxycoumarin-3-carboxylic acid amino-D-alanine (HADA), a fluorescent derivative of D-alanine, we are able to capture where and when PG synthesis occurs^51^. Co-incubation of HADA with top-performing β-lactams would not only validate the activity at the cellular level, but may provide insights into the mechanism of action in *T. pallidum* PG synthesis. We first incubated *T. pallidum* cultures with 0.5 mM HADA for 24 h and compared signal intensities to diluent-fed, control cells. HADA signal was near ubiquitous throughout the cell and the intensity was variable, but significantly higher than DMSO control-treated cells (Fig. 5a, b). We next characterized the spatiotemporal dynamics of PG synthesis in a population of cells using a demograph^52^. Demographs determine the relative fluorescent signal intensity about the long-axis of the cell in a population that has been organized based on length. The shortest cells in this analysis are those at the beginning of the cell cycle (i.e., newly divided), while the longest are approaching the end of the cycle and are poised for division. Population-level analysis (*n* = 414) indicated near ubiquitous incorporation of PG along the sacculi (Fig. 5c, d, stage I). Towards the end of the *T. pallidum* cell cycle, HADA signal increased at the mid-cell site, indicative of septum formation (Fig. 5c, d, stage II). After dividing, vestiges of the septum were apparent at the poles of the bacterium (Fig. 5c, d, stage III). This mechanism of PG synthesis is consistent with so-called lateral growth, which is common among rod-shaped bacteria and reminiscent of the closely related spirochete *Treponema denticola*^38,53^.

To determine how and/or if selected β-lactams impact *T. pallidum* PG synthesis at the cellular level, we co-incubated cultures with HADA and each compound at a final concentration of 2 times the MIC, as determined previously for each (Fig. 4). All treatments resulted in increased HADA signal, which was unexpected (data not shown). One explanation could be that treated cells were actively importing an analog of D-alanine from their environment, which is to be expected of bacterial scavengers like *T. pallidum*, but failed to incorporate the molecule into their cell wall due to treatment resulting in increased HADA signal localized to the cytoplasm. We reasoned that this scenario could be circumvented by analyzing purified PG. By boiling treated cells in 10% sodium dodecyl sulfate, unincorporated HADA, and virtually all other cellular components, are solubilized, and insoluble sacculi can be analyzed. To validate this approach, we first performed the same demograph analysis as above, but on SDS insoluble, *T. pallidum* sacculi (Fig. 5e). PG-derived HADA signal was highly similar to intact cells (Fig. 5c, e). This comparative study also uncovered that sacculi are ~2–4 µm shorter than intact cells, which is consistent with the apparent polar space seen in cryo-electron micrographs^53^. We next proceeded to analyze the total fluorescence HADA signal intensity present in sacculi that had been pre-treated with the refined list of β-lactams described above. Indeed, purified PG contained significantly lower HADA-derived signal for all β-lactam treated samples, indicating that each was effective at preventing PG biosynthesis (Fig. 5f).

## Discussion

Since the first recorded outbreak in the late 15th century, syphilis has caused severe morbidity and mortality worldwide. The emergence of penicillin in the 1940s helped curtail disease complications and lowered death rates^7,11,54^. Recent surges in cases, coupled with antibiotic supply shortages, drug allergies, and resistance to certain macrolide antibiotics have energized the interest in new therapies to treat syphilis^22^. Clinical and experimental animal studies are limited in the number of possible treatment options that can be tested and directly compared. The advent of a prolonged in vitro cultivation system has provided new avenues of *T. pallidum* research. We took advantage of this system to provide a comprehensive, basic science approach, to directly compare nearly a hundred pre-approved β-lactams for use in humans. Our studies uncovered several compounds that may be appropriate to treat syphilis in a clinical setting. We discuss the potential implications herein, as well as in the broader context of *T. pallidum* biology.

Benzathine penicillin G is currently the clinical β-lactam of choice for treating syphilis and is administered intramuscularly as one dose for early stages and three doses delivered over three weeks for tertiary syphilis^22^. Benzathine penicillin G (also called benzathine penicillin) is two monomers of penicillin G complexed with diphenylethylene diamine (Fig. 4). The former is the active component and collectively satisfies the two major requirements for β-lactams efficacy. The latter satisfies the other major requirement of improving stability and half-life of the active component, from approximately 0.5 h to 6 h in the case of benzathine penicillin G^55,56^. While the severity and degree of cross-reactivity of penicillin allergies vary greatly, the R_1_ group (C7 position) is the primary culprit associated with the vast majority of penicillin allergies meaning many individuals can tolerate other β-lactams^57–59^. In addition to being non-reactive in penicillin-sensitive patients and having a long half-life, new syphilis treatment options would need to be effective at all disease stages. Currently, only multiple high doses of benzathine penicillin G is used for treating late-stage syphilis infections; in neurosyphilis penicillin G or procaine penicillin is used^22^. The biological basis of tertiary syphilis is not well understood but numerous accounts of the use of benzathine penicillin G to ameliorate or recover from debilitating symptoms provide a strong argument for the use of antibiotics in these instances^60–66^. The high degree of efficacy at low nanomolar concentrations of the compounds, with several compounds having lower MICs compared to benzathine penicillin G, make them strong candidates for future in vivo studies (Table 1).

It is unclear how well different β-lactams may perform in a clinical setting. But, from the many compounds tested, at least 23 could prevent bacterial growth as indicated by relative efficacy at 5 nM concentration (Figs. 2–4) and a few have even been partially validated through clinical trials^22^. Our iterative sampling approach of β-lactams led to the identification of four penicillin derivatives with MICs ranging from 0.5–1.2 nM (Fig. 4). Mezlocillin and azlocillin were identified as the two β-lactams with the lowest MIC for in vitro cultivated *T. pallidum* (Fig. 4). Comparative structural similarity analysis predicts that neither would cause an allergic response in penicillin sensitive individuals^67^. Unfortunately, both are no longer produced in the United States. Nafcillin, on the other hand, (1) was very similar to the MIC of benzathine penicillin G; (2) contains a structurally distinct sub-group from benzathine penicillin G; and (3) costs less than other options (Table 1). We note, however, that the cost analysis we provide (Table 1) requires additional considerations for context. First, each value represents bulk pricing and may not reflect patient cost in a clinical care setting. It is also important to consider the number of doses required and the route of administration, which may vary with each treatment. These caveats notwithstanding, Nafcillin is widely available due to its efficacy in treating penicillinase-producing bacteria like *Staphylococcal* and *Streptococcal* species^68^. The latter is noteworthy since the only enzymatically characterized *T. pallidum* PBP, Tp47, is also a penicillinase^44,45^.

The four other β-lactams we identified as being highly active against *T. pallidum* were cephalosporins, albeit to a lesser extent than the penicillin group (MIC 1.6–5 nM, Fig. 4 and Table 1). Cephalosporins differ from penicillins in that they carry a dihydrothiazine core and can possess an additional R-group (i.e., R_1_ (C7 position) and R_2_ (C3)). In this group was ceftriaxone, whose relative efficacy was assessed against *T. pallidum* in vitro prior to the advent of a continuous in vitro culture system, with very similar results^69^. Ceftriaxone has recently garnered considerable attention as an alternative to benzathine penicillin G^70,71^; it also has the added advantages of having both an extremely long half-life and a high degree of penetrance into the central nervous system (CNS) (Table 1)^72^. Cefmenoxime has the same R_1_ group as ceftriaxone (2-(2-amino-1,3-thiazol-4-yl)-2-(methoxyimino)acetylamino), is also capable of circulating in the CNS^73^, and is among the cheapest of the compounds tested (Table 1). The remaining cephalosporins—cefmetazole and cefazedone—along with those above, all carry R_1_ groups that reduce the likelihood of IgE or T-cell mediated responses in penicillin-allergic patients^25,58,59,74,75^.

Aside from identifying possible candidates for future clinical consideration, our studies provide insights into *T. pallidum* biology. All organisms tightly regulate their cell length and *T. pallidum* is no exception (Fig. 1). Despite being cultured in non-axenic growth conditions, in a dish, the population-level cell length (13.31 µm ±3.51) is remarkably similar to bacteria in vivo^40^. Our work has shown that many of the advances in the field of *Borreliae* cell biology, such as quantitative microscopy and automated cell detection, can now be applied to study the syphilis spirochete, despite the dramatic differences in helical pitch (Figs. 1 and 5)^38,76–79^. These tools were paramount in determining that unlike the Lyme and relapsing fever *Borrelia*, which elongate by synthesizing heritable zones of PG, *T. pallidum* cell wall synthesis is lateral (Fig. 5). In this way *T. pallidum* PG synthesis is akin to *T. denticola*^38^. Another feature that we discovered here that differentiates these genera is the overall morphology of the PG. Pure *B. burgdorferi* PG is a straight, elongated tube that is not coiled^38,80^. The flat-wave morphology of *B. burgdorferi* is dictated entirely by the ribbon-like flagellar filaments that wrap around the flexible PG^80,81^. *T. pallidum* PG, on the other hand, appears to maintain some coiled features despite being treated with protease (Supplementary Figure 1). It is tempting to speculate that the intermediate filament protein CfpA, produced by many species of treponemes, may be involved in contorting the cell wall, similar to an unrelated intermediate filament protein in *Leptospira interrogans*^82–84^.

A wealth of *T. pallidum* knowledge has been produced despite the experimental challenges attributed to a lack of a stable in vitro culture system. This frontier has passed and brought about a new age of research, which made this work possible. While our findings will require animal model and clinical validation, we have provided a shortlist of strong candidates to expand syphilis therapeutics. We hope that our studies provide motivation to consider and/or potentially repurpose existing β-lactams for instances when the current recommended therapeutic strategy isn’t appropriate or possible.

## Methods

### *T. pallidum* in vitro culture

All studies were performed on an isolate of *T. pallidum*, sub-species *pallidum*, strain Nichols that was provided by Steve Norris and Diane Edmondson. They also provided the immortalized Sf1Ep rabbit epithelial cells required for *T. pallidum* cultivation. Sf1Ep cells were propagated in MEME (Sigma #M5650) supplemented with 10% heat-inactivated FBS, 2 mM L-glutamine, and 0.75 mM sodium pyruvate at 37 °C with 5% CO_2_. Cells were used between passage 13 and 36. The day before passaging *T. pallidum*, Sf1Ep cells were seeded into a 12-well plate at a density of 4 × 10^4^ per well. Additionally, TpCM2 was prepared and left to equilibrate overnight in a microaerophilic incubator at 37 °C with 5% CO_2_, 1.5% O_2_ and 93.5% N_2_ as described previously^14^.

### Antibiotic susceptibility testing

A frozen stock from previous in vitro lab cultivation was used to initiate the continuous culture which was maintained for six weeks prior to the study start date. Four to 6 hours prior to passaging, Sf1Ep media was removed from plates seeded with Sf1Ep cells, the plates were washed once with 0.5 mL of equilibrated TpCM2, and the media was replaced with 2 mL of TpCM2. The antibiotic panel included all 89 cell wall targeting compounds found in the ‘Antibiotic compound library’ (Product nL-5300), produced by Selleckchem LLC (Houston, TX). For reference purposes, we also included tetracycline and doxycycline from the library/provider. Each antibiotic was added to fresh media at a concentration of 5 nM solubilized in either water or DMSO. A positive control with just water or DMSO was used as a comparison. *T. pallidum* (1 × 10^6^ cells) were passaged into each well in a 12-well plate from the established culture. After 1 week all media was collected from each well, the well was washed once with 0.175 mL trypsin EDTA, then treated with 0.175 mL trypsin EDTA for five minutes at 37 °C. The media, trypsin EDTA rinse, and trypsinized culture were combined and diluted 1:10 in sterile PBS and enumerated under a dark field microscope (Nikon E600, 40X LWD objective) using a Fuchs-Rosenthal disposable hemocytometer (iNCYTO #DHC-F01). Together, this optical approach can detect ~1000 cells per milliliter of culture media. All conditions were enumerated three times and averages were reported; visible spirochetes were considered when determining culture density.

MIC was defined as the concentration of compounds that caused the culture to be less than or equal to the inoculum and determined via microscopic enumeration using the same procedure as above. Compounds were tested at concentrations from 0.125 to 8 nM and each drug concentration was tested in duplicate. The mean and SD were calculated from the replicates and were used to construct a modified Gompertz regression from which the MIC was interpolated^85^.

### RNA isolation and qRT-PCR

*T. pallidum* cultures were exposed to drug compounds for one week and collected as described above. Cultures were centrifuged at 4000 × *g* for 15 min at 4 °C, supernatant was removed, and cells were immediately processed for RNA using the Zymo Research Quick-RNA miniprep kit. To ensure no DNA contamination samples were subjected to an additional DNAse1 treatment with 50 units of RNase free, recombinant DNase1 (Sigma #04716728001). Quantitative RT-PCR was performed on processed RNA samples from *T. pallidum* treated with compounds using the Luna universal one-step RT-qPCR kit (New England BioLabs #E3005L). An annealing step of 50 °C for 10 seconds was added to the cycle protocol to account for the T_m_ of the primer sets. Quantification of RNA was done by targeting both the proposed PBP gene, *tp47*, and a flagellar sheath protein, *flaA*. Both primer sets were constructed by Integrated DNA Technologies and have been previously validated elsewhere^17,86^.

**Table:** 

tp47	forward	(5’ -TCA ACC GTG TAC TCA GTG -3’)
	reverse	(5’ - CGT GTG GTA TCA ACT ATG G - 3’)
*flaA*	forward	(5’ - AAC GGA GTC GAA CAG GAG ATA C 3’)
	reverse	(5’ - CGT GTG GTA TCA ACT ATG G - 3’)

### HADA Labeling

*T. pallidum* was cultured as described above and allowed to grow for four days. HADA was added to cultures for a final concentration of 0.5 mM with a volume/volume ratio of DMSO. After 24 h cultures were trypsinized and collected, as described above, and fixed with 1.8% paraformaldehyde while rocking for 5 min. Samples were left on ice for 20 min to stop the fixation reaction before being centrifuged at 4000 × *g* for 10 min at 4 °C and washed with sterile PBS three times. Finally, samples underwent one additional spin of 4000 × *g* for 15 min at 4 °C before being resuspended in <200 µL sterile PBS and transferred to amber tubes to be stored at 4 °C until imaging.

For HADA labeling of drug-treated cells, *T. pallidum* subcultures were allowed to grow for three days before the addition of the selected antibiotic at a concentration of two times the calculated MIC. After 24 h of antibiotic exposure, subcultures were pulse-labeled with HADA at a final concentration of 0.5 mM with a volume/volume ratio of DMSO. After 24 h cultures were trypsinized and collected as described above before being centrifuged at 4000 × *g* for 15 min. The supernatant was removed, and the pellet was resuspended in 500 µL of sterile PBS before being added dropwise to warm 10% sodium dodecyl sulfate solubilized in sterile water in amber tubes. The cell suspension-SDS mixture was heated at 100°C for one hour to isolate the peptidoglycan. The tubes were centrifuged at 20,000 × *g* for 30 min at 23 °C to pellet the purified sacculi. The supernatant was promptly removed, and the pellet was washed with sterile, ultrapure water (Apex #18-193). This was repeated for a total of three washes. The final pellet was resuspended in less than 50 µL of sterile ultrapure water and stored at room temperature until imaging.

To protease treat sacculi, isolated sacculi were incubated with 100 µL of 3 mg/mL chymotrypsin at 37 °C, shaking at 540 rpm for 18 h.

### Microscopy

Imaging of HADA-labeled fixed whole cells and unfixed purified sacculi was done using phase-contrast and epifluorescence microscopy. Whole cells and isolated sacculi were imaged on a 2% agarose pad on a Zeiss Axio Observer using an oil-immersion phase-contrast Plan Apochromat 100x/1.45 numerical aperture objective (Nikon) using a Hamamatsu Ocra-Flash 4.0 V3 Digital CMOS camera. Phase contrast imaging had a typical exposure time of 10 ms. Epifluorescence imaging utilized DAPI channel with an excitation wavelength of 360 nm and an emission wavelength of 397 nm and a typical exposure time of 500 ms. Image acquisition occurred over multiple weeks and was determined to be reproducible so multiple days of imaging were pooled for population-level analysis.

Image analysis was performed using Oufti, an automated cell detection software that has previously been used on other spirochetes. Values were obtained for >400 cells or sacculi for control populations and >100 sacculi for drug-treated populations. Parameters were modified to allow for *T. pallidum* whole cell and isolated sacculi detection and can be found in the supplemental information. Total signal intensity was normalized to cell length for comparison using custom MATLAB scripts which can be found in Supplementary Information.

### Reporting summary

Further information on research design is available in the Nature Research Reporting Summary linked to this article.

## Supplementary information

Supplementary Information

REPORTING SUMMARY

## Data Availability

All data reported in the manuscript are either represented in the figures or in Supplementary Information.

## Code availability

All scripts used to analyze or depict the data can be found in Supplementary Information.
