# Supplementary material for: A large screen identifies beta-lactam antibiotics which can be repurposed to target the syphilis agent
Source: NPJ Antimicrob Resist. 2023 Jun 1;1:4. doi: 10.1038/s44259-023-00006-3 (PMC11057208; doi:10.1038/s44259-023-00006-3)
Supplement: Supplementary file 1 — Supplementary Information [file 44259_2023_6_MOESM1_ESM.pdf]

A large screen identifies beta-lactam antibiotics which can be repurposed to target the syphilis agent

Hayes *et al*, 2023

Supplementary Information: Table of Contents

Page 1	Table of contents
Page 2-3	Supplementary Table 1
Page 4	Supplementary Figure 1–HADA labeled sacculi pre- and post-protease treatment
Page 5-7	Oufti parameters for *T. pallidum*
Page 7-9	MATlab script for normHistOutline
Page 9	MATlab script for signal intensity normalized by length

Supplementary Table 1. All antimicrobial compounds tested in this study

CLASS	NAME	TYPE
**Penicillin**	Aztreonam	Monobactam
	Cloxacillin sodium	Penicillinase-resistant
	Amoxicillin sodium	Aminopenicllin
	Amoxicillin	Aminopenicllin
	Ampicillin sodium	Aminopenicllin
	Carbenicillin disodium	Extended spectrum
	Ticarcillin sodium[*]	Extended spectrum
	Azlocillin sodium salt[*]	Extended spectrum
	Nafcillin sodium[*]	Penicillinase-resistant
	Dicloxacillin sodium	Penicillinase-resistant
	Ampicillin trihydrate	Aminopenicllin
	Penicillin G sodium[*]	Natural
	Mezlocillin sodium[*]	Ampicillin derived
	Piperacillin sodium[*]	Extended spectrum
	Faropenem sodium	Penems
	Flucloxacillin sodium	Penicillinase-resistant
	Benzylpenicillin potassium[*]	Natural
	Amoxicillin trihydrate	Aminopenicllin
	Sulbenicillin sodium	Other
	Sultamicillin	Other
	Pivmecillinam hydrochloride	Other
	Penicillin G procaine[*]	Natural
	Doripenem	Penems
	Ertapenem sodium	Penems
	Benzathine penicillin[*]	Natural
	Oxacillin sodium	Penicillinase-resistant
	Procaine penicillin G[*]	Natural
	Dicloxacillin sodium hydrate	Penicillinase-resistant
	Amdinocillin	Other
	Oxacillin sodium monohydrate[†]	Penicillinase-resistant
	Penicillin V potassium salt[†]	Natural
	6-aminopenicillanic acid[†]	Other
**Cephalosporin**	**NAME**	**GENERATION**
	Cefaclor	2
	Cefdinir	3
	Cefoperazone[*]	3
	Cefditoren pivoxil[*]	3
	Ceftiofur HCl	3
	Ceftibuten dihydrate	3
	Cefpirome sulfate	4
	Cefamandole nafate	2
	Cefepime dihydrochloride monohydrate	4
	Cefsulodin sodium	3
	Cefonicid sodium	2
	Cefotaxime sodium	3
	Cephalothin	1

Cefazolin sodium	1
Cefixime	3
Cefuroxime sodium	2
Cefmenoxime hydrochloride[*]	3
Ceftizoxime[*]	3
Cefuroxime axetil	2
Cefoxitin sodium[*]	2
Cefcapene pivoxil hydrochloride	3
Cefetamet pivoxil hydrochloride	3
Cefodizime sodium	3
Cefazedone[*]	1
Cephapirin benzathine	1
Cefathiamidine	3
Ceforanide	3
Ceftezole	2
Cephalotin acid	1
Ceftezole sodium	2
Cefquinome sulfate	4
Cefpodoxime proxetil	3
Cefmetazole sodium[*]	2
Cefminox sodium	2
Cefpiramide sodium[*]	3
Ceftiofur	3
Cefotiam Hexetil hydrochloride	3
Ceftizoxime sodium[*]	3
Cephapirin sodium	1
Cefotaxime	3
Cefoperazone sodium[*]	3
Cefazolin	1
Cefoxitin[*]	2
Cefozopran hydrochloride	4
Ceftaroline fosamil	5
Cefodizime	3
Ceftiofur sodium	3
Ceftriaxone sodium[*]	3
Ceftazidime[†]	3
Ceftriaxone sodium trihydrate[*, †]	3
Cefradine[†]	1
Cephradine monohydrate[†]	1
Latamoxef sodium[†]	3
Cefoselis Sulfate[†]	4
7-Aminocephalosporanic acid[†]	Functional group
Cefadroxil hydrate[†]	1
Cephalexin[†]	1

All compounds came from the same lot/library preparation, purchased from Sellack Inc. Results were compared to doxycycline and tetracycline, which came from the same library. All compounds were tested at a final concentration of 5 nM, diluted in DMSO (0.1% final), unless otherwise noted by those diluted in water(†). * Top 25% of compounds tested.

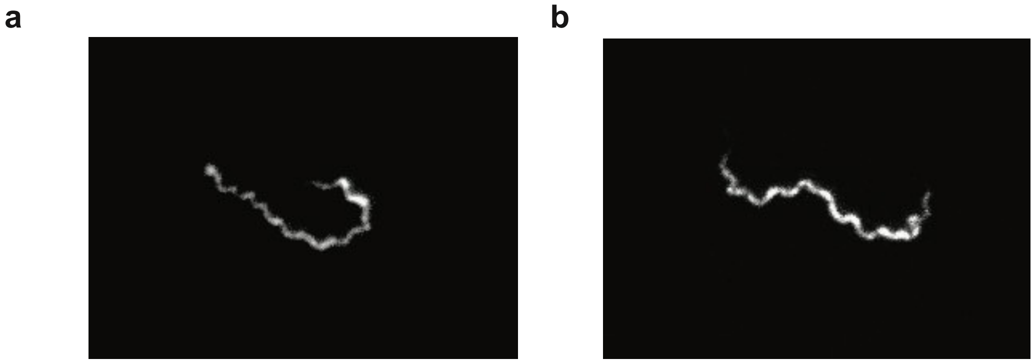

Supplementary Figure 1. Purified sacculi from HADA labeled *T. pallidum*. a, *T. pallidum* bacteria were pulse labeled with 0.5mM HADA for 24 hours and boiled in 10% SDS to isolate the sacculi. **b,** Isolated sacculi from HADA labeled *T. pallidum* postchymotrypsin treatment. Scale bar = 5 um.

Oufti parameters for *T. pallidum*

% This file contains Oufti suggested parameters for Treponema pallidum %cells
at 0.064 um/pixel resolution.

%High-throughput
outCsvFormat = 0
csvFileEdit = 0 runSerial
= 0

%Parallel Computation maxWorkers
= 12

%General algorithm
= subpixel
invertimage = 0
interpoutline = 0
interSigma = 0

% Pixel-based parameters
areaMin = 800 areaMax =
30000 splitregions = 1
displayW = 0 wShedNum
= 5800

%Constraints (alg. subpixel only)
cellwidth = 8 wspringconst = 0.2
rigidity = 0 rigidityB = 2

% Image forces (alg. subpixel)
imageforce = 7 attrCoeff =
0.25 repCoeff = 0.9 neighRep
= 8 attrPower = 8

%Contour fitting (alg. subpixel)
fitDisplay = 0
fitMaxIter = 150 moveall = 0.4
fitStep = 0.2 fitStepM = 1
fitCondition = 0
%Mesh Creation
fsmooth = 240
roiBorder = 22.5
noCellBorder = 1
meshStep = 1 getmesh
= 1

% Joining and splitting
splitThreshold = 1

joindist = 5
joinangle = 0.2
joindilate = 1

%parameters added after Segmentation Module
edgemode = 1 erodeNum = 2 openNum = 3
invertimage = 0 thresFactorM = 0.21557
thresFactorF = 0.21557 threshminlevel = 0
edgeSigmaL = 3.6701 valleythresh1 = 0.0001
logthresh = -0.56286
% This file contains Oufti suggested parameters for Treponema pallidum
%cells at 0.064 um/pixel resolution.

%High-throughput
outCsvFormat = 0 csvFileEdit
= 0
runSerial = 0

%Parallel Computation
maxWorkers = 12

%General algorithm
= subpixel
invertimage = 0
interpoutline = 0
interSigma = 0

% Pixel-based parameters
areaMin = 800 areaMax =
30000 splitregions = 1
displayW = 0 wShedNum
= 5800

%Constraints (alg. subpixel only)
cellwidth = 7.5 wspringconst =
0.2 rigidity = 0
rigidityB = 2

% Image forces (alg. subpixel)
imageforce = 7 attrCoeff =
0.25 repCoeff = 0.9 neighRep
= 8
attrPower = 8

%Contour fitting (alg. subpixel)
fitDisplay = 0 fitMaxIter = 150

moveall = 0.4 fitStep = 0.2
fitStepM = 1
fitCondition = 0

%Mesh Creation
fsmooth = 240
roiBorder = 22.5
noCellBorder = 1
meshStep = 1 getmesh
= 1

% Joining and splitting
splitThreshold = 1
joindist = 5
joinangle = 0.2
joindilate = 1

%parameters added after Segmentation Module
edgemode = 1 erodeNum = 2 openNum = 3
invertimage = 0 thresFactorM = 0.21557
thresFactorF = 0.21557 threshminlevel = 0
edgeSigmaL = 3.6701 valleythresh1 = 0.0001
logthresh = -0.56286

MATlab script for normHistOutline

function[varargout] = normHistOutline(ints,bins,rgb)
%normHistOutline plots a histogram normalized to area = 1 of hdata if %either rgb is specified or if output arguments are not collected.
%
%Acceptable use:
%In general,
%[x, y] = normHistOutline(...)
% Returns x and y for plotting, a plot will be created only if rgb is specified
%normHistOutline(...)
% If no output is collected, a plot will be displayed
%
%normHistOutline(hdata,rgb)
% plots a normalized histogram with color rgb
%normHistOutline(hdata,bins,rgb)
% bins hdata in n bins and plots in color rgb
%normHistOutline(hdata,bins)
% bins hdata in n bins and plots in default color (black)
%
%hdata can be a vector or matrix of any shape to be plotted
%

```
%bins is an optional argument specifying the number of segments hdata
%should be split into. if not specified, bins will be set to the square
%root of length of hdata
%
%rgb is a 3 element vector bounded from 0 to 1 specifying the color to
%plot. alternatively, rgb can be a standard matlab color symbol (see
%colorspec)

if nargin == 0
   %if no arguements are provided, return a useage example
hold on
   normHistOutline((-1.9+.9*randn(2000,1)),'r')
normHistOutline((.7*randn(2000,1)),'g')
normHistOutline((2.1+.5*randn(2000,1)),'b')
return end

if nargin == 1
   bins = floor(sqrt(length(ints)));
   rgb = [0 0 0]; end

%if a color was provided in the place of bins, set bins to the default
%value and set rgb accordingly
if length(bins) == 3 || ischar(bins)
   rgb = bins;     bins =
floor(sqrt(length(ints)));
end

%convert hdata into an n by 1 matrix ints
= ints(:);
%use built-in hist function to calculate histogram over bins
[y,x] = hist(ints,bins);
%normalize area of the histogram to 1
y = y/sum(y.*diff([min(ints) x(1:end - 1) + diff(x)/2 max(ints)]));

binsz = mean(diff(x));
%calculate the outlines of the histogram in x
x           = cat(1,x-
binsz/2,x,x+binsz/2); x
= x(:)'; x = [x(1), x,
x(end)];
%calculate the outlines of the histogram in y
y           = cat(1,y,y,y); y
= y(:)'; y = [0,y,0];

if nargout == 0 ||nargin == 3     if
~exist('rgb'),rgb = [0 0 0];end
plot(x,y,'Color',rgb,'LineWidth',1.2)
set(gca,'Box','on','FontSize',16)
ylabel('Frequency')     xlabel('Event')
elseif nargout == 2     varargout{1} =
x;     varargout{2} = y; end end
```

```
cellList = oufti_makeCellListDouble(cellList);
for ii = 1:length(cellList.meshData)     for jj =
1:length(cellList.meshData{ii})
       cellList.meshData{ii}{jj} = getextradata(cellList.meshData{ii}{jj});
end end
```

MATlab script for signal intensity normalized by length

```
counter = 1; for frames = 1:length(cellList.meshData)     for cells =
1:length(cellList.meshData{frames})         if
~isempty(cellList.meshData{frames}{cells}.signal1)
meanSignal1(counter) = mean(cellList.meshData{frames}{cells}.signal1);
sumSignal1(counter) = sum(cellList.meshData{frames}{cells}.signal1);
signal1AreaNorm(counter) =
sum(cellList.meshData{frames}{cells}.signal1)/cellList.meshData{frames}{cells}.area;
counter = counter + 1;
       end
end end

figure
normHistOutline(meanSignal1)
xlabel('Average Pixel Intensity per Cell')

figure
normHistOutline(signal1AreaNorm)
xlabel({'Summed Pixel Intensity','Normalized by Cell Area (px)'})

figure
normHistOutline(sumSignal1) xlabel({'Summed
Pixel Intensity per Cell'})
```
